# Clinical Remarks on Typhoid Pneumonia

**Published:** 1847-05

**Authors:** Greneau De Meussy


*Clinical Remarks on Typhoid Pneumonia.* By Greneau de Meussy.—The occurrence of a case of fever in the wards of Hôtel Dieu, in which a previously unsuspected pneumonia was discovered after death, gave rise to the following remarks:—

Let us inquire what is the difference between primary pneumonia, that is to say, pneumonia occurring without any other appreciable disease, and secondary or consecutive pneumonia. Consecutive pneumonia is generally a most insidious disease, and arises and makes considerable progress without giving rise to any particular symptom. The initiatory rigor, so much insisted upon by pathologists, as well as the pain in the side, is usually either entirely wanting, or is so slight that it escapes the attention of the physician. Dyspnœa, greater than is to be accounted for by the febrile condition of the patient, is in general the only rational sign which is presented.

Nevertheless, by auscultation we shall find either the crepitous râle, or, if the disease is more advanced, bronchial respiration and bronchophony; we must, therefore, in doubtful cases, have immediate recourse to the stethoscope, if we would recognize the disease at its commencement. But there are certain cases, of which the present is an example, in which the greatest attention to the physical condition of the chest fails to detect any abnormal sound; in these cases it is probable that the pneumonia is central, and will only be recognized as the inflammation extends to the surface.

In some instances the invasion of pneumonia is announced by a sudden increase of prostration, with an alteration in the features expressive of anxiety, and emaciation. However full the pulse may have previously been, it becomes small and thready. There is also occasionally quiet delirium, or a state of adynamic somnolence, which shows itself suddenly during the progress of the fever. This is the form of pneumonia to which authors have applied the term *latent*, but the term is in truth misapplied; for though certain symptoms are absent, the auscultatory sounds are in most cases as marked as in the ordinary idiopathic disease. What we have said in reference to the pain applies also to all those symptoms for the manifestation of which a certain amount of sensibility and vital power is necessary. The cough and expectoration, for example, are less marked in patients who are delirious or extremely exhausted, and the physician is in such cases deprived of all valuable diagnostic signs.

It must be stated also, that it is rare to find the physical signs of typhoid or secondary pneumonia as distinctly defined as in the idiopathic form of the disease. For instance, the crepitous râle is composed of larger and less equal bubbles, and is often masked by other râles, as the sibilous or sonorous; a valuable diagnostic sign exists in the extraordinary heat of the skin, and in a general increase of febrile symptoms.

In speaking of the treatment of this adynamic form of pneumonia, the lecturer remarks upon the impossibility of having recourse to depletory measures, and the necessity of caution in the use of the ordinary internal remedies for pneumonia, as tartar emetic, in consequence of the irritable, or inflamed, or ulcerated condition of the alimentary canal sometimes present in these cases. He veiy properly relies chiefly upon counter-irritation, with the guarded exhibition of tartar emetic in small doses.

The lecturer omits to mention a very necessary part of the management of these severe cases, namely, the exhibition of nourishment, and in some cases of stimulants.—*Gazette des Hôpitaux.—Ibid.*

